# A Light-Weight Text Summarization System for Fast Access to Medical Evidence

**DOI:** 10.3389/fdgth.2020.585559

**Published:** 2020-12-04

**Authors:** Abeed Sarker, Yuan-Chi Yang, Mohammed Ali Al-Garadi, Aamir Abbas

**Affiliations:** ^1^Department of Biomedical Informatics, School of Medicine, Emory University, Atlanta, GA, United States; ^2^Department of Biomedical Engineering, Georgia Institute of Technology and Emory University, Atlanta, GA, United States; ^3^Heinz College of Information Systems and Public Policy, Carnegie Mellon University, Pittsburgh, PA, United States

**Keywords:** medical text processing, text summarization, text mining, natural language processing, health informatics, extractive summarization

## Abstract

As the volume of published medical research continues to grow rapidly, staying up-to-date with the best-available research evidence regarding specific topics is becoming an increasingly challenging problem for medical experts and researchers. The current COVID19 pandemic is a good example of a topic on which research evidence is rapidly evolving. Automatic query-focused text summarization approaches may help researchers to swiftly review research evidence by presenting salient and query-relevant information from newly-published articles in a condensed manner. Typical medical text summarization approaches require domain knowledge, and the performances of such systems rely on resource-heavy medical domain-specific knowledge sources and pre-processing methods (e.g., text classification) for deriving semantic information. Consequently, these systems are often difficult to speedily customize, extend, or deploy in low-resource settings, and they are often operationally slow. In this paper, we propose a fast and simple extractive summarization approach that can be easily deployed and run, and may thus aid medical experts and researchers obtain fast access to the latest research evidence. At runtime, our system utilizes similarity measurements derived from pre-trained medical domain-specific word embeddings in addition to simple features, rather than computationally-expensive pre-processing and resource-heavy knowledge bases. Automatic evaluation using ROUGE—a summary evaluation tool—on a public dataset for evidence-based medicine shows that our system's performance, despite the simple implementation, is statistically comparable with the state-of-the-art. Extrinsic manual evaluation based on recently-released COVID19 articles demonstrates that the summarizer performance is close to human agreement, which is generally low, for extractive summarization.

## Introduction

The overarching objective of evidence-based medicine practice is to actively incorporate the best available and most reliable scientific evidence into clinical practice guidelines and decision-making (1). The movement associated with the establishment of evidence-based medicine practice has led to the development of evidence hierarchies for medical research, establishment of clinical practice guidelines, and recognition of the importance of patient-oriented evidence (2, 3). Since the inception of the modern concept of evidence-based medicine, medical practitioners have been advised to combine their clinical expertise and understanding of patients' priorities with the latest scientific evidence (4–6). Early and recent studies have extensively discussed the problem of *information overload* that many practitioners face, particularly in clinical settings, due to the massive amounts of research evidence that is available and the continuous growth of such evidence (7). Searching through medical evidence regarding a specific topic is time-consuming, and practitioners often consider the task to be unproductive and futile (8–10). PubMed^1^, which indexes over 30 million articles, typically returns multiple pages of research publications even when the queries are very targeted and specific. Almost two decades ago, Hersh et al. (11) discussed the long time (30 min, on average) that it takes for experienced practitioners to search for evidence, and, particularly at point-of-care, practitioners cannot afford to spend that much time. Over time, with the increasing rate of publication of medical literature, these problems associated with evidence curation have only increased (12). Improved literature searching and fast access to relevant and summarized information can be particularly beneficial for medical students and young practitioners because of their lack of clinical experience, or at times when there is a burst of growth in research evidence on a topic (e.g., the ongoing COVID19 pandemic).

Natural language processing (NLP) and information retrieval methods have the potential to aid medical experts and researchers to collect and review the latest and emerging research evidence in an efficient manner. NLP methods can, for example, help experts formulate effective search queries and summarize individual publications. Query-focused text summarization approaches have specifically been explored to aid medical practitioners adhere to evidence-based medicine principles (13–15). These systems take queries (in natural language or key-terms) as input and generate/extract the query-relevant summaries. In terms of automatic summary quality, the performances of successful approaches designed for the medical domain have relied heavily on domain-specific knowledge sources (16). For example, the pioneering work by Demner-Fushman and Lin (17) incorporated sentence-level knowledge in a supervised classification system trained to detect *outcome* sentences, which were regarded as summary sentences. Sarker et al. (14) and ShafieiBavani et al. (15) utilized manually annotated summarization datasets to generate extractive and abstractive summaries—both systems relying heavily on the identification of domain-specific generalizations, concepts, and associations. Similarly, Hristovski et al. (18) proposed the use of domain-specific semantic relations for performing question answering for biomedical literature. Building on past research progress, recent studies have proposed end-to-end question-answering systems, which typically contain modules to perform the summarization (12, 19). Such systems, however, are generally only suitable for very specific types of queries, and despite their limited scopes, they invariably require the incorporation of medical domain-specific knowledge sources. The progress of summarization and question-answering research in the medical domain has been relatively sluggish, requiring considerable amounts of research efforts to overcome each of the many hurdles. Further discussion of the chronological progress in this research space is outside the scope of this brief research report, and detailed descriptions of medical domain-independent and domain-specific text summarization systems over the years are available through survey papers (20–22).

Adaptation of summarization systems to a particular domain can be computationally expensive and require large numbers of external tools (23). Within the medical domain, systems typically attempt to incorporate domain knowledge based on the Unified Medical Language System *via* software such as MetaMap (24), which can tag lexical representations of medical concepts. This is in turn used in downstream tasks, or as features in learning systems. Heavy dependence on these domain-specific systems introduces disadvantages, some of which are as follows:

- the systems are not very portable or generalizable, and are only suitable for the very specific tasks they were initially designed and evaluated for;
- they are difficult to re-implement and/or deploy without the domain-specific knowledge sources or ontologies; and
- they are computationally slow, often un-parallelizable.

The goal of our work is to design a resource-light and fast medical text summarization system that is decoupled from domain-specific knowledge sources. This work is an extension of our years of past research on this topic, focusing specifically on operational and deployment simplicity. The proposed system is extractive and query-focused in design. It relies on publicly available labeled data, which is used for weight optimization, unlabeled data—specifically, dense word embeddings learned from the unlabeled data—and a set of simple features that require little computational resources and time. In the development and evaluation processes, we selectively added and removed modules based on their performance and resource requirements. Comparative evaluation of our system against a state-of-the-art system on a standard dataset showed that it is capable of generating summaries of comparable qualities, despite its simplistic design.

## Methods

The primary dataset for this research is a corpus specifically for NLP research to support evidence-based medicine, created by Molla-Aliod et al. (25) with the involvement of the first author of this paper. The specialized corpus contains a total of 456 queries along with expert-authored single- and multi-document evidence-based summarized responses to them. Each query is generally associated with multiple single-document summaries, which present evidence from distinct studies. The abstracts of the studies from which the answers were derived are made available from PubMed. In total, the corpus contains 2,707 single-document summaries. To ensure fair comparisons, we used the exact train-test split from past research (14)−1,388 for training and 1,319 for evaluation. The system we compare against is very reliant on domain-specific NLP resources, and it had produced state-of-the-art performance on the described corpus. The second dataset is much smaller, and we had prepared it to manually evaluate the performance of the summarization system. This dataset consists of a small set of articles describing research potentially relevant to COVID19. For each of these included articles, we manually created extractive summaries in response to a standard query, and we compared the agreement between our system and the manual summaries.

For developing and optimizing the system, we used the training set to devise feature scoring methods and learn weights for all the feature scores. Here, the training set does not consist of the exact single-document summaries, which are abstractive summaries authored by human experts. Instead, the training set consists of three-sentence extractive summaries for each document so that the gold standard is consistent with the expected output of our summarizer. These three-sentence extractive summaries of the training set are generated by computing the ROUGE-L F_1_-score of all three-sentence combinations against the human summary, and selecting the top-scoring sentence combination for each text. We chose three as our target number of sentences based on past research (14, 17).

During the summary generation process, each sentence from the full set of candidate summary sentences receives a score for each feature included in the summarization system. All candidate sentences are then scored as the sum of the weighted feature scores, and the three sentences with the highest scores are extracted as the summary. The scoring process takes into consideration the target sentence position, the sentence length and the contents of the selected sentences. In the final summary, the selected sentences are presented sequentially (from first to last). The scoring process can be summarized as:


$$\begin{array}{l} \zeta_{m,t_{n}=\sum_{i=1}^{k}\left(w_{i,m,n}\times f_{i,m,n}|S_{m},t_{n}\right)} \end{array}$$

where ζ_*m*,_*t*__*n*__is the score for sentence number *m* of a text, given the summary target sentence number *t*_*n*_, and *w*_*i,m,n*_ and *f*_*i,m,n*_ are the weight and score for feature *i*, respectively. For each summary sentence position (*t*_*n*_), the top-scoring sentence is chosen. To explore and discover a set of *simple* but *salient* features, we started with the full set of features used by the QSpec system and removed modules or features with the highest dependencies and longest running times. For example, one important derived feature in the QSpec system is a sentence-level score based on the *sentence type*, the *UMLS semantic types* present in the sentence, and the *associations between semantic types*. Identifying the sentence type requires the execution of an automatic classifier at run-time (26), identifying UMLS semantic types and associations requires the execution of MetaMap (24), and once these processes are completed, an exhaustive concept-level search is performed to find and score the sentence based on the presence of each association. Due to the computational complexity of this module, and its dependence on external tools, we removed this feature first and attempted to optimize performance using the other features—those attempted in the past and those we added. In addition to the features used for the QSpec system, we evaluated a number of features such as variants of edit-distance-based lexical similarities and scores based on the presence of possible statistical testing information (e.g., *p*-values). These features did not contribute to meaningfully improve the overall system score, and they were also eventually excluded. Following experimentation with multiple feature combinations, we selected five that could be computed fast and proved to be useful when used in combination. We describe these features in the following paragraphs.

### Word Embedding-Based Maximal Marginal Relevance

Maximal Marginal Relevance (MMR) (27) is a strategy that can be used to increase relevance and reduce redundancy, and variants of it have been popular for text summarization (28–31). In our approach, we compute two similarity measures—between sentences and the associated query, and between the sentences themselves. During score generation, sentences are rewarded for being similar to the query, while at the same time they are penalized for being similar to sentences that have already been selected to be included in the summary. The similarity values are combined linearly with suitable weights (λ):


$$\begin{array}{l} MMR=\lambda \times SIM\left(S_{m},Q\right)-\left(1-\lambda \right)\times \underset{S_{c}\epsilon S_{sel}}{\max}\left(SIM\left(S_{m},S_{c}\right)\right) \end{array}$$

where *SIM*(*S*_*m*_, *Q*) is the similarity score between a sentence and the query and $\underset{S_{c}\epsilon S_{sel}}{\max}\left(SIM\left(S_{m},S_{c}\right)\right)$ is the maximum similarity between the same sentence and the set of already-selected summary sentences. Choosing the best three-sentence summary is a combinatorial optimization problem, and MMR enables us to approach sentence selection in a sequential manner. Despite the widespread use of MMR for extractive summarization, two variants of this score that we use in this system, which rely on distributed representations of the words in the sentences and the queries, had not been proposed in the past, to the best of our knowledge. We obtained pre-trained embeddings that were generated from all PubMed and PubMed Central (PMC) Open Access texts (32) using the *word2vec* tool^2^ (vector size = 200, window size = 5) and the *skip-gram* model (33). For the first variant, we compute the similarity between two text segments (i.e., sentence vs. query and sentence vs. sentence) as the *average* cosine similarity of all the terms. We compute this average by adding the cosine similarities of all the term combinations and dividing by the product of the lengths of the two texts. For the second variant, we use the word vectors in a text segment to compute its *centroid* in vector space. A single centroid is computed for the set of all words within the set of already-chosen sentences (*S*_*sel*_). These centroids are then used to compute MMR.

### Traditional MMR Score

For the traditional MMR score, the third variant used in the system, we first pre-processed the terms by lowercasing, stemming and removing stop words. We then computed the *tf* × *isf* for each word in a sentence and the query—where *tf* is the frequency of a term in a text segment and *isf* is the *inverse sentence frequency* of the term in all the texts (i.e., the inverse of how many sentences, including the query, contain the term). We then generated vectors for each sentence using the *tf* × *isf* values of the terms.

### Sentence Length Score

Sentence length is a metric that may filter out uninformative, short sentences by assigning them a lower score, while rewarding sentences that are relatively longer in a document. In summarization tasks where the character lengths of the summaries are limited, longer sentences may also be penalized (34, 35). We attempted to assign penalties to very short sentences (e.g., 1−3-word sentences), which often represent section headers. At the same time, our goal was to assign higher scores to longer sentences—with decreasing gradients for very long sentences, such that this score does not play a significant role in choosing between those informative sentences.

**Figure 1:**
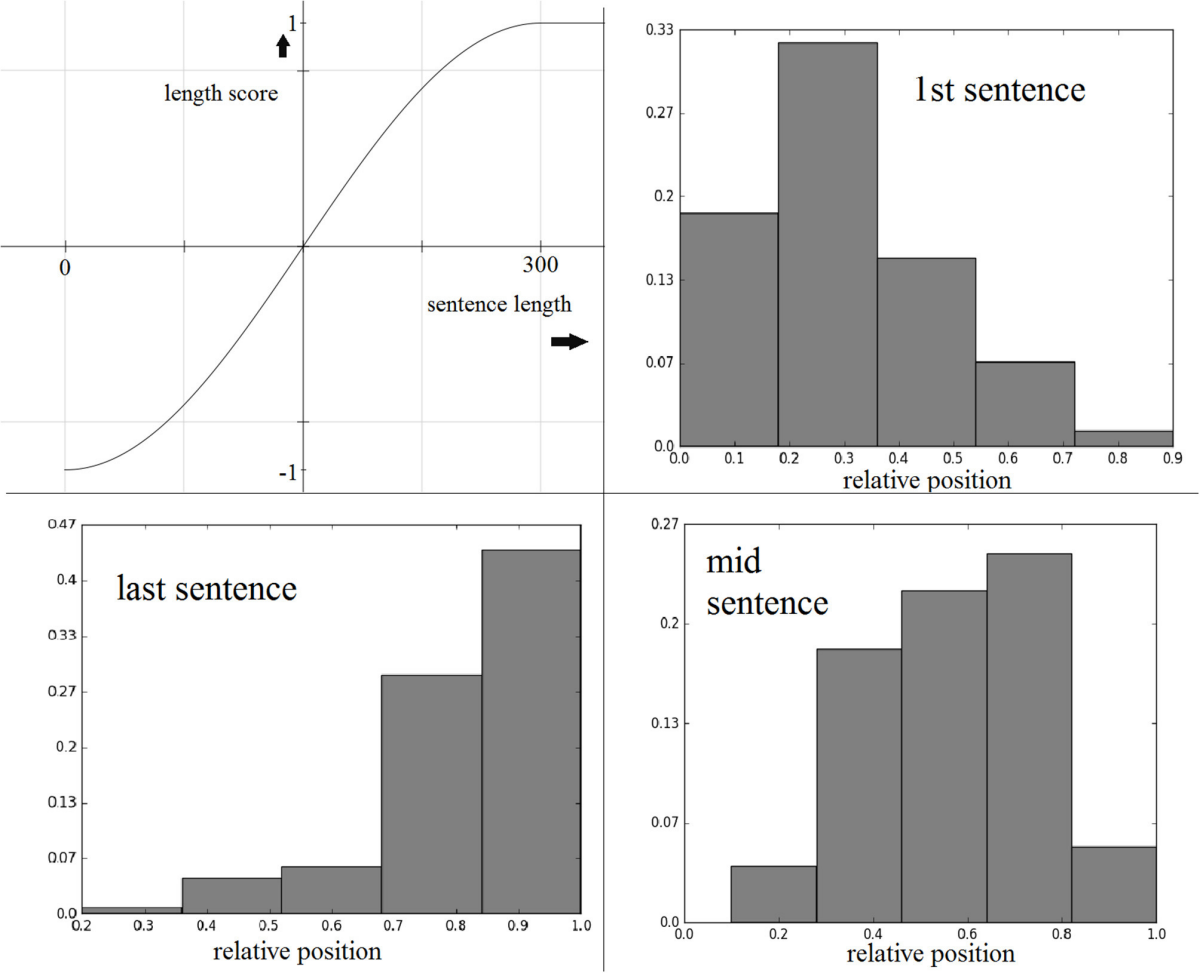
Clockwise from top-left: sine function for sentence length score, maxed at 300 characters; first, middle, and last sentence relative position distributions from the best-scoring extractive training set summaries.

Our experiments on the training set suggested that a *sinusoidal* function conveniently served this purpose. The average sentence length in the training data is ~150 characters, so we considered 0 and 300 characters to be the lower and upper length limits, respectively, and mapped the lengths to the range $\left(\frac{-\pi }{2},\frac{\pi }{2}\right)$. Following that, we applied a *sin* function to the mapped value to generate a length score between (−1, 1). Figure 1 illustrates how a sin function enables us to reward/penalize sentences based on their lengths relative to the average sentence length. Both reward and penalty start to level off as length approaches 0 or 300.

### Sentence Position Score

Our last score is based on sentence position and the target sentence number. Sentence position has been shown to be a crucial metric for extractive summarization in domains including news (36) and medical (17). We used an approach identical to our past work as it had proven to be computationally fast and effective (14). The approach, which we called *target sentence specific summarization*, generates different scores for the same source sentence based on the summary sentence number. This means that the same sentence gets a different score when the system is searching for the first sentence for a three-sentence summary compared to when the system is searching for the last sentence. This ensures that the eventual summary extract is not biased to a specific region of the source text, which is often the case with traditional systems that apply the same scoring mechanism for all text spans. Generally speaking, when the system is scoring sentences for the first summary sentence, it gives preference to sentences occurring early in the source documents, which often contain important background information, compared to sentences occurring later, which tend to contain information about the final outcome of the study.

To compute this score, we first obtained the best three-sentence summary (gold standard summary) for each training text, and used these sentences to generate normalized frequency distributions of the relative sentence positions. These distributions are shown in Figure 1. During summary generation, given the relative sentence position *r* of a source sentence, the score assigned is the normalized frequency for *r* in the given target sentence distribution.

### Weight Optimization and Intrinsic Evaluation

We computed near-optimal weights for scoring using the training set *via* a grid search in the range (0.0, 1.0). For each weight combination, all the three-sentence training set summaries were generated and the weights producing the highest F_1_-score were used for evaluation on the test set. The ROUGE summary evaluation tool (37) was used to compare the extractive summaries with the expert-authored summaries in the corpus. The ROUGE-L variant of the evaluation tool attempts to score summaries based on their longest common subsequences (LCS) (38). Given two texts—the automatic summary of length *m* words and the corresponding gold-standard summary of length *n* words—the F_1_-score is computed as:


$$\begin{array}{l} F_{1}-score=\frac{\left(1+\beta^{2}\right)\times R\times P}{\left(R+\left(\beta^{2}\;\times P\right)\right)} \end{array}$$

where *R* = *LCS*(*summary, goldstadard*)/*m*; *P* = *LCS*(*summary*, *goldstandard*)/*n* and *LCS*(*summary, goldstandard*) is the length of the longest common subsequence between the summary and the gold standard. β^2^ is set to 1. ROUGE scores had been shown to be correlated with human evaluators and the ROUGE-L F_1_-score is the harmonic mean of the ROUGE-L recall and precision scores. In past research, the evaluations of many summarization systems were based on summaries constrained by character-level maximum lengths (e.g., 100 characters), and such evaluations typically used ROUGE recall scores for comparison. In our case, the summaries are constrained by the number of sentences (three), and so, optimizing and evaluating based on recall would overfit the system in favor of longer sentences. Therefore, we chose to use the F_1_-score, rather than recall, and we computed them using the original human-authored summaries as the gold standard.

### Extrinsic Evaluation on COVID19 Literature

We conducted a brief extrinsic evaluation of the system using a small number of recently-published articles about COVID19 or related research (39). We created six categories of queries focusing on different types of COVID19-related information (e.g., *treatment* and *transmission*). To establish these categories, we selected 2 from 12 categories that had been proposed in the literature (40) and added 4 additional ones. Two of our query categories (*treatment* and *prognosis*) overlapped with the categories proposed in the past, and we added 4 more categories based on their relevance to COVID19 and our research interests. Note that our intent was not to determine a comprehensive set of categories relevant to COVID19. The queries, their types and their numbers are shown in Table 1. For a set of 11 articles we manually created 3-sentence summaries. The four authors independently created the three-sentence summary for each article. We modeled the sentence selection task as a binary sentence labeling task and compared the pair-wise agreements between the annotators using Cohen's kappa (41).

**Table 1:** Queries used for extrinsic evaluations, their types and numbers.

**Query**	**Type**	**Number annotated**
What measures can be taken to lower the transmission of COVID-19?	Transmission	3
What are some of the mental health impacts of COVID19?	Mental health	3
What is the common prognosis for COVID19 infection?	Prognosis	2
What treatment is effective for COVID19	Treatment	1
What are the common risks of COVID19 on targeted populations?	Risks	1
How does the impact of COVID19 vary based on social inequalities?	Inequalities/economic	1

We ran the summarizer with the best performing weights on a large amount of COVID19 literature that has been made available since the outbreak of the pandemic. For 11 of these articles, which were manually annotated, we compared the agreements between the three-sentence human summaries, and between the system and human summaries. We also compared the agreement between the human summaries and summaries generated by the QSpec system. Two authors were kept unaware of the internal scoring strategy of the system to ensure that sentence selection is not biased by that knowledge. Note that the articles themselves were pre-selected, and were not based on the queries, since information retrieval is not an objective of our research.

## Evaluation and Results

### Automatic Evaluations

Table 2 presents the performance of our system along with several other systems. Identical training-test splits were used for evaluation. Our proposed approach obtains a score of 0.166, 0.002 lower than the best-performing system. The table shows that despite the simplicity of our approach, its performance is comparable to the state-of-the-art, and significantly better than other baselines. To compare our approach with the extractive summarization method proposed by Demner-Fushman and Lin (17), we used an automatic classifier to detect *Outcome* sentences (42); the last three outcome sentences were extracted as the summary. Using the ROUGE score distribution of all summary combinations, we computed the percentile rank of our summarizer's performance *via* the method described by Ceylan et al. (43). In the proposed approach, a probability density function is generated using an exhaustive search of all ROUGE score combinations for extractive summaries, and this distribution is used to find the percentile rank of a system's ROUGE score. Our light-weight system's score has a percentile rank of 94.3 compared to QSpec's rank of 96.8. The large difference in percentile rank despite the small change in the ROUGE score is caused by the typical long-tailed nature of the ROUGE score distribution. The computed optimal weights for the features were: sentence position weight = 0.8; sentence length: 0.2; MMR (traditional): 0.2; MMR (dense vectors; both variants): 0.5.

**Table 2:** Comparison of ROUGE-L F_1_-scores for our summarizer with other systems and 95% confidence intervals.

**System**	**ROUGE-L F_**1**_ Score**	**95% CI**
Our system	0.166	0.162–0.170
QSpec (14)	0.168	0.164–0.172
Last 3 Sentences	0.155	0.151–0.158
Demner-Fushman and Lin (17)	0.159	0.152–0.164
Random	0.154	0.150–0.157
First 3 Sentences	0.140	0.136–0.143

### Extrinsic Summary Evaluation

Pair-wise agreement, based on Cohen's kappa, was generally low for both sets of agreements (i.e., human-human and human-system). Table 3 presents the average system-human, all human-human, and subsets of human-human agreements. Sample human and automatic summaries are provided in Supplementary Material. A link to the final human summaries, after resolving disagreements, are also provided in the Supplementary Material.

**Table 3:** Average agreements between the system and human annotators, all human annotators and subsets.

**Author/system**	**Kappa (average)**
Human-system agreement average	0.33
Human-QSpec agreement average	0.35
Human-human agreement average	0.40
Average: annotators 1, 2 and 3	0.41
Average: annotators 1, 2 and 4	0.40
Average: annotators 1, 3 and 4	0.34
Average: annotators 2, 3 and 4	0.38

## Discussion and Conclusion

**Figure 2:**
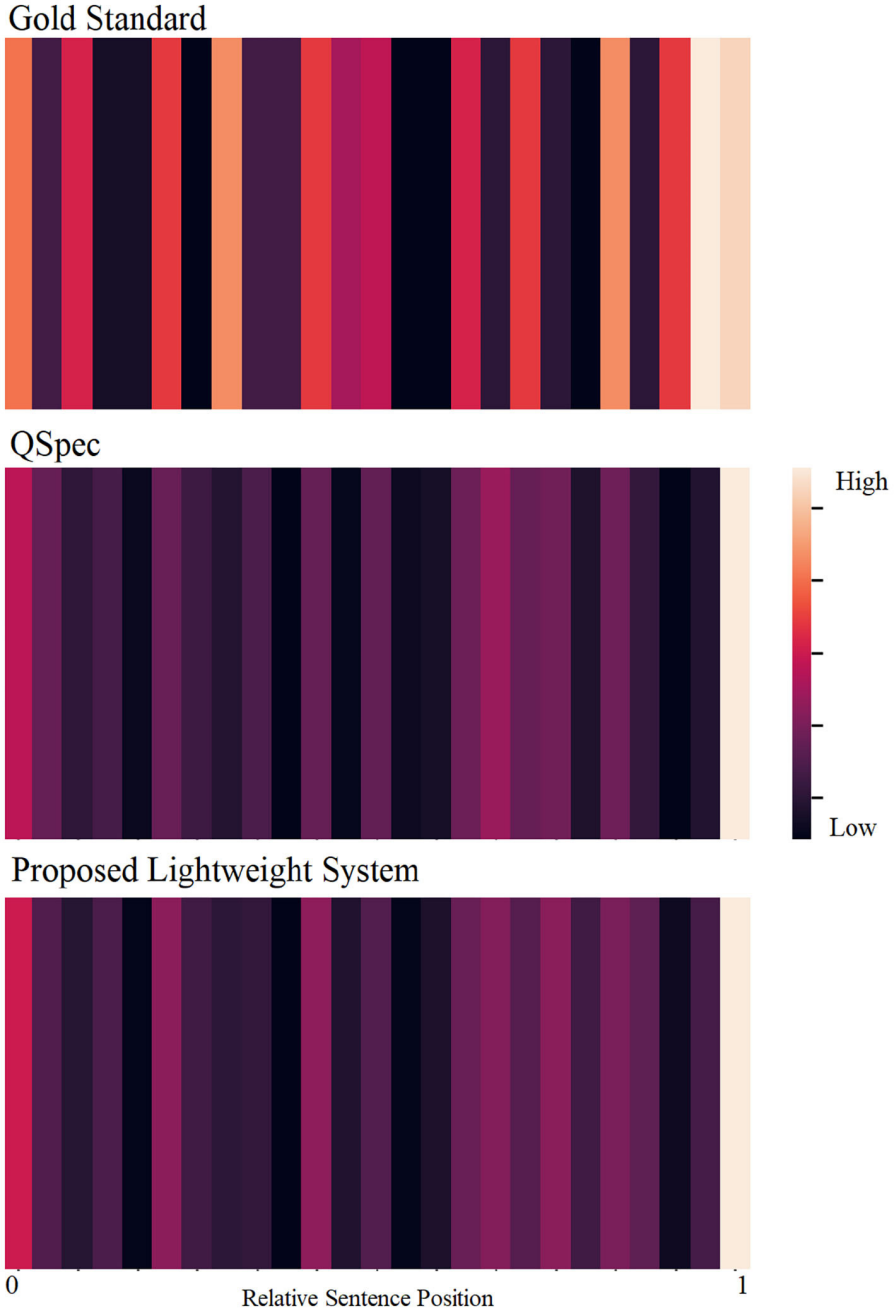
Distributions of relative sentence positions of the gold standard summary sentences (top), and automatically-generated summaries (QSpec: middle; proposed system: bottom).

Using a set of similarity-based and structural features, our system performs comparably to the state-of-the-art system, with a ROUGE-L F_1_-score of 0.166. Our extrinsic evaluations showed that for this extractive summarization task, human-to-human inter-annotator agreement was low, resulting in a low ceiling for the automatic summarizer. We observed consistently low agreements across subsets of annotators, illustrating that choosing the optimal n-sentence query-focused summary is a difficult task for humans. Abstractive summaries could perhaps be more suitable for humans as more information can be summarized within a short text span. However, from the perspective of automatic summarization, moving from extractive to abstractive summarization has been challenging for this particular research community, and our scope was limited to extractive summarization. Although our evaluation was brief and differences between automatic and human summaries were not conclusive, we did observe more disagreements for earlier summary sentence selections compared to the selection of later sentences. Generally speaking, we found the gold standard summaries to have higher variance in relative sentence positions compared to automatically generated summaries. Figure 2 further illustrates the differences between the gold standard extracts and the automatic summarization systems by visualizing the distributions of the relative positions of the sentences included in the summary. While human summaries almost invariably contain sentences from the end of the texts, they also tend to contain sentences from different relative positions. However, QSpec and our proposed system tend to select most sentences from the end and some from the beginning, but few from the rest of the document.

Our specific focus for this summarization system was to make the sentence selection process simple and decoupled from multiple additional systems or processes while also maintaining high performance. Our focus on simplicity is particularly from the perspective of deploy-ability (i.e., *how quickly can the source for the system be downloaded and executed on a new machine?*). Past systems focusing on the task of evidence-based medicine text summarization have relied on multiple knowledge-encapsulating software sources such as MetaMap, and parallel processes such as query and sentence classification, Compared to the resource-heavy QSpec system, which requires query and sentence classification and the generation of UMLS semantic types/associations, our current approach requires minimal pre-processing. Only a set of pre-trained word embeddings are required. The light-weight nature of the summarizer also means that it runs faster than QSpec. On a standard computer (Intel® i5 2.0 GHz processor), it takes our summarizer a few minutes to summarize all the documents in the test set. Due to the simplicity of our approach, we believe that it can be easily re-implemented, customized or extended for real-life settings, and the results can be reproduced without requiring the use of third-party tools. It is possible for non-NLP experts or even non-programmers to use the summarization system without having to set up additional tools; the only resource needed is any publicly available pre-trained word/phrase embedding model.

From an application perspective, we believe that this summarization approach is more easily *transferable* to other data sets, even those in other languages that do not have domain knowledge encoded in thesauruses. Exploring the applicability of this approach on non-English datasets is part of our future research plans. We are particularly interested in assessing the performance of this system, compared to those reliant on domain-specific knowledge sources, on other languages without including a language-specific gold standard or manually-curated knowledge sources. Our hypothesis is that this light-weight summarizer will outperform resource-heavy systems such as QSpec on such data sets.

We obtained the word embeddings from past research and used them without modification. There is a possibility that the learning of these embeddings can be customized to the summarization task for improving performance (e.g., using a COVID19-specific embedding model for the second summarization task). This is a notable limitation of the system—the semantics of emerging health topics, such as COVID19, may not be captured by the underlying embedding model, thus, leading to sub-optimal performance. Another operational limitation may be the size of the embedding model. While our focus is on a light-weight system that can be run on not-so-powerful computers, embedding models can be large in size (multiple gigabytes), which may act as an obstacle for old machines. To address these limitations, in future research, we plan to implement a continuously-learning embedding model that updates periodically using text from recently-published papers, and strategies for building targeted embedding models that require less unlabeled data and memory at run time.

## Data Availability Statement

Publicly available datasets were analyzed in this study. This data can be found here: https://sourceforge.net/projects/ebmsumcorpus/ and https://sarkerlab.org/lw_summ/.

## Author Contributions

AS implemented the initial system and evaluated. AA, MA-G, and Y-CY assisted with the experiments, evaluations, and in preparing the manuscript. All authors contributed to the article and approved the submitted version.

## Conflict of Interest

The authors declare that the research was conducted in the absence of any commercial or financial relationships that could be construed as a potential conflict of interest.

## Footnotes

^1^https://www.ncbi.nlm.nih.gov/pubmed/ (accessed 25 Nov, 2019).

^2^https://code.google.com/p/word2vec/ (accessed 17 Nov, 2019).
