# Supplementary material for: A Light-Weight Text Summarization System for Fast Access to Medical Evidence
Source: Front Digit Health. 2020 Dec 4;2:585559. doi: 10.3389/fdgth.2020.585559 (PMC8521877; doi:10.3389/fdgth.2020.585559)
Supplement: Supplementary file 1 [file Data_Sheet_1.PDF]

Supplementary Material

1 Sample Summaries

In the following summaries, we show the queries, the title of each article, and two sample manual summaries.

EXAMPLE 1

Query: What measures can be taken to lower the transmission of COVID-19?

Title: Spread and Impact of COVID-19 in China: A Systematic Review and Synthesis of Predictions From Transmission-Dynamic Models [1]

PMID: 32626719

Automatic Summary:

We searched PubMed, Web of science, EMBASE, bioRxiv, medRxiv, arXiv, Preprints, and National Knowledge Infrastructure (Chinese database) for relevant studies published between 1 December 2019 and 21 February 2020.
The overall median basic reproduction number (R(0)) was 3.77 [interquartile range (IQR) 2.78–5.13], which dropped to a controlled reproduction number (R(c)) of 1.88 (IQR 1.41–2.24) after city lockdown.
Although interventions in China have been effective in controlling secondary transmission, sustained global efforts are needed to contain an emerging pandemic.

Manual Summary 1:

We searched PubMed, Web of science, EMBASE, bioRxiv, medRxiv, arXiv, Preprints, and National Knowledge Infrastructure (Chinese database) for relevant studies published between 1 December 2019 and 21 February 2020.
The overall median basic reproduction number (R(0)) was 3.77 [interquartile range (IQR) 2.78–5.13], which dropped to a controlled reproduction number (R(c)) of 1.88 (IQR 1.41–2.24) after city lockdown.
Our analysis found a sustained R_c and prolonged incubation/ infectious periods, suggesting COVID-19 is highly infectious.

Manual Summary 2:

Fifty-two articles involving 75 mathematical or statistical models were included in our systematic review.
The overall median basic reproduction number (R0) was 3.77 [interquartile range (IQR) 2.78–5.13], which dropped to a controlled reproduction number (Rc) of 1.88 (IQR 1.41–2.24) after city lockdown.
Although interventions in China have been effective in controlling secondary transmission, sustained global efforts are needed to contain an emerging pandemic.

EXAMPLE 2

Query: What are some of the mental health impacts of COVID19?

Title: Structuring mental health support for frontline caregivers during COVID-19: lessons from organisational scholarship on unit-aligned support [2]

PMCID: PMC7299658

Automatic Summary:

This article translates organisational scholarship on professionals working in organisations to elucidate why traditional approaches to supporting employee mental health, which often ask employees to seek assistance from centralised resources that separate mental health personnel from frontline units, may be insufficient under crisis conditions.
We describe how an intentional organisational design used by the US Army that assigned specific mental health personnel to frontline units helped to mitigate professional goal and identity conflicts by creating personalised relationships and contextualising mental health offerings.
Addressing frontline caregivers' mental health needs is a vital part of health delivery organisations' response to COVID-19, but without thoughtful organisational design, well-intentioned efforts may fall short.

Manual Summary 1:

To address these pervasive goal and identity conflicts in professional organisations, we translate the results of a multiyear research study examining the US Army's efforts to transform its mental health support during the wars in Iraq and Afghanistan.
We describe how an intentional organisational design used by the US Army that assigned specific mental health personnel to frontline units helped to mitigate professional goal and identity conflicts by creating personalised relationships and contextualising mental health offerings.
Addressing frontline caregivers' mental health needs is a vital part of health delivery organisations' response to COVID-19, but without thoughtful organisational design, well-intentioned efforts may fall short.

Manual Summary 2:

This article translates organisational scholarship on professionals working in organisations to elucidate why traditional approaches to supporting employee mental health, which often ask employees to seek assistance from centralised resources that separate mental health personnel from frontline units, may be insufficient under crisis conditions.
We highlight parallels between providing support to frontline military units and frontline healthcare units during COVID-19 and surface implications for structuring mental health supports during a crisis.
Addressing frontline caregivers' mental health needs is a vital part of health delivery organisations' response to COVID-19, but without thoughtful organisational design, well-intentioned efforts may fall short.

EXAMPLE 3

Query: What is the common prognosis for COVID-19 infection?

Title: Leucocyte Subsets Effectively Predict the Clinical Outcome of Patients With COVID-19 Pneumonia: A Retrospective Case-Control Study [3]

PMID: 32626680

Automatic Summary:

Older age, elevated aspartate aminotransferase, total bilirubin, serum lactate dehydrogenase, blood urea nitrogen, prothrombin time, D-dimer, Procalcitonin, and C-reactive protein levels, decreased albumin, elevated serum cytokines (IL2R, IL6, IL8, IL10, and TNF-α) levels, and a decreased lymphocyte count indicated poor outcome in patients with COVID-19 pneumonia.
Lymphocyte subset (lymphocytes, T cells, helper T cells, suppressor T cells, natural killer cells, T cells+B cells+NK cells) counts were positively associated with clinical outcome (AUC: 0.777; AUC: 0.925; AUC: 0.900; AUC: 0.902; AUC: 0.877; AUC: 0.918, resp.).
These results indicate that leucocyte subsets predict the clinical outcome of patients with COVID-19 pneumonia with high efficiency.

Manual Summary 1:

Hospitalized COVID-19 pneumonia patients with definitive clinical outcome (cured or died) were retrospectively studied.
Older age, elevated aspartate aminotransferase, total bilirubin, serum lactate dehydrogenase, blood urea nitrogen, prothrombin time, D-dimer, Procalcitonin, and C-reactive protein levels, decreased albumin, elevated serum cytokines (IL2R, IL6, IL8, IL10, and TNF-α) levels, and a decreased lymphocyte count indicated poor outcome in patients with COVID-19 pneumonia.
These results indicate that leucocyte subsets predict the clinical outcome of patients with COVID-19 pneumonia with high efficiency.

Manual Summary 2:

Lymphocyte subset (lymphocytes, T cells, helper T cells, suppressor T cells, natural killer cells, T cells+B cells+NK cells) counts were positively associated with clinical outcome (AUC: 0.777; AUC: 0.925; AUC: 0.900; AUC: 0.902; AUC: 0.877; AUC: 0.918, resp.).
The neutrophil-to-lymphocyte ratio (NLR), neutrophil to T lymphocyte count ratio (NTR), neutrophil percentage to T lymphocyte ratio (NpTR) effectively predicted mortality (AUC: 0.900; AUC: 0.905; AUC: 0.932, resp.).
Binary logistic regression showed that NpTR was an independent prognostic factor for mortality.

EXAMPLE 4

Query: What treatment is effective for covid?

Title: Whole Genome Analysis and Targeted Drug Discovery Using Computational Methods and High Throughput Screening Tools for Emerged Novel Coronavirus (2019-nCoV) [4]

PMID: 32617527

Automatic Summary:

A novel coronavirus designated as SARS-CoV-2 in February 2020 by World Health organization (WHO) was identified as main cause of SARS like pneumonia cases in Wuhan city in Hubei Province of China at the end of 2019.
Molecular docking results against protein targets Furin, papain like proteases, RdRp and Spike glycoprotein had shown paritaprevir, ritonavir, entecavir and chloroquine derivatives are the best drugs to inhibit multi targets of coronavirus infection including natural compounds corosolic acid, baicalin and glycyrrhizic acid with minimal inhibitory concentrations.
Thus we propose use of paritaprevir, entecavir, ritonavir and chloroquine derivatives as best drug combination along with niacinamide, folic acid and zinc supplements to treat novel coronavirus infection.

Manual Summary 1:

Currently, there is no specific treatment against the new virus.
Thus we propose use of paritaprevir, entecavir, ritonavir and chloroquine derivatives as best drug combination along with niacinamide, folic acid and zinc supplements to treat novel coronavirus infection.
We also propose use of plant protease inhibitors (PI's) and Anti-IL8, IL-6, IL-2 as future drug models against coronavirus.

Manual Summary 2:

Molecular docking results against protein targets Furin, papain like proteases, RdRp and Spike glycoprotein had shown paritaprevir, ritonavir, entecavir and chloroquine derivatives are the best drugs to inhibit multi targets of coronavirus infection including natural compounds corosolic acid, baicalin and glycyrrhizic acid with minimal inhibitory concentrations.
Thus we propose use of paritaprevir, entecavir, ritonavir and chloroquine derivatives as best drug combination along with niacinamide, folic acid and zinc supplements to treat novel coronavirus infection.
We also propose use of plant protease inhibitors (PI's) and Anti-IL8, IL-6, IL-2 as future drug models against coronavirus.

Gold standard summaries:

The finalized, manually-created gold-standard summaries are available here:
https://sarkerlab.org/lw_summ/

REFERENCES

1. Lin YF, Duan Q, Zhou Y, et al. Spread and Impact of COVID-19 in China: A Systematic Review and Synthesis of Predictions From Transmission-Dynamic Models. Front Med (Lausanne). 2020;7:321. Published 2020 Jun 18. doi:10.3389/fmed.2020.00321.
2. DiBenigno J, Kerrissey M. Structuring mental health support for frontline caregivers during COVID-19: lessons from organisational scholarship on unit-aligned support. BMJ Leader. 2020 Jun:leader-2020-000279. doi: 10.1136/leader-2020-000279. Epub 2020 Jun 2. PMCID: PMC7299658.
3. Gan J, Li J, Li S, Yang C. Leucocyte Subsets Effectively Predict the Clinical Outcome of Patients With COVID-19 Pneumonia: A Retrospective Case-Control Study. Front Public Health. 2020 Jun 18;8:299. doi: 10.3389/fpubh.2020.00299. PMID: 32626680; PMCID: PMC7314901.
4. Manikyam HK, Joshi SK. Whole Genome Analysis and Targeted Drug Discovery Using Computational Methods and High Throughput Screening Tools for Emerged Novel Coronavirus (2019-nCoV). J Pharm Drug Res. 2020;3(2):341-361. Epub 2020 Mar 30. PMID: 32617527; PMCID: PMC7331973.
